# Genome-Wide Association Analysis and Gene Mining of Resistance to China Race 1 of Frogeye Leaf Spot in Soybean

**DOI:** 10.3389/fpls.2022.867713

**Published:** 2022-06-22

**Authors:** Maolin Sun, Chen Na, Yan Jing, Zhihui Cui, Na Li, Yuhang Zhan, Weili Teng, Yongguang Li, Wenbin Li, Xue Zhao, Yingpeng Han

**Affiliations:** ^1^Key Laboratory of Soybean Biology in Chinese Ministry of Education, Key Laboratory of Soybean Biology and Breeding, Genetics of Chinese Agriculture Ministry, Northeast Agricultural University, Harbin, China; ^2^College of Tropical Crops, Hainan University, Haikou, China

**Keywords:** soybean, frogeye leaf spot, resistance locus, GWAS, beneficial alleles, candidate genes

## Abstract

Soybean frogeye leaf spot (FLS) is a worldwide fungal disease. Its higher occurrence frequency and wider distribution range always led to severe yield losses of soybean, therefore, breeding new cultivars with FLS resistance has been an important breeding goal for soybean breeders. In this study, an association panel of 183 representative soybean accessions was used to evaluate their resistance to FLS race 1, and to identify quantitative trait nucleotides (QTNs) and candidate genes based on genome-wide association study (GWAS) and high-throughput single-nucleotide polymorphisms (SNPs). A total of 23,156 high-quality SNPs were developed using the specific locus-amplified fragment sequencing (SLAF-seq) approach. Finally, 13 novel association signals associated with FLS race 1 resistance were identified by the compressed mixed linear model (CMLM). In addition, 119 candidate genes were found within the 200-kb flanking genomic region of these 13 peak SNPs. Based on the gene-based association analysis, haplotype analysis, expression pattern analysis, and virus-induced gene silencing (VIGS) systems, four genes (*Glyma.05G121100*, *Glyma.17G228300*, *Glyma.19G006900*, and *Glyma.19G008700*) were preliminarily proved to play an important role in the soybean resistance to FLS race 1.

## Introduction

Frogeye leaf spot (FLS) of soybean [*Glycine max* (L.) Merrill], caused by *Cercospora sojina Hara*, was reported in Japan in 1915 for the first time (Mian et al., 2008; Gu et al., 2020). Subsequently, FLS were identified in warm and humid soybean-growing regions including North America, China, and Africa (Mian et al., 2008; Soares et al., 2015). The pathogen populations of FLS was complicated and included many types of physiological races. Different researchers identified 11 races in America, 15 races in China, and more than 20 races in Brazil based on specific differential host system, respectively (Gu et al., 2020). For these 15 FLS races in China, race 1 was the main race (Ding et al., 2012). Though FLS could infect seed, pods, and stems, typical symptoms of FLS developed primarily on foliage of soybean in the V3 stage and led to a premature defoliation (Fehr and Caviness, 1977; Mengistu et al., 2019). FLS was a polycyclic disease throughout the growing season of soybean, therefore, FLS easily outbreak and caused severe epidemics in warm and humid environment (Kim et al., 2013). Akem and Dashiell (1994) reported that FLS epidemics could cause yield reduction of soybean greater than 60%. Though FLS could be controlled by planting disease-free seed, treatment of seed with a fungicide before planting, crop rotation, and treatment of R2–R5 growth stage soybean foliage with fungicides, cultivar with resistance was still most effective to manage this disease (Mian et al., 2008; Kim et al., 2013). Management of FLS has primarily relied on single dominant resistance genes known as *Rcs* genes. *Rcs1* from ‘Lincoln,’ resistant to American race 1, was reported in 1952 for the first time (Athow and Probst, 1952). Subsequently, *Rcs2*, resistant to American race 2, was identified from ‘Kent’ (Probst et al., 1965). *Rcs3*, the most durable and robust gene, was found in ‘Davis’ and exhibited the resistance not only to race 5, but also to all other known isolates of *C. sojina* in the United States and Brazil (Yorinori, 1992; Mengistu et al., 2012). Though some dominant genes were found from different resistance sources, breeding resistance cultivars through traditional method based on the phenotypic evaluation is still inefficient and time-consuming.

Marker-assisted selection (MAS) can increase efficiency of traditional selection method through improving the allele’s frequency of desirable FLS quantitative trait loci (QTL). To date, only few molecular markers for FLS have been reported. SSR marker Satt244, located on Chromosome (Chr.) 16 (linkage group, LG) was reported to be within 1 centimorgan (cM) of the *Rcs3* gene (Mian et al., 1999). The *Rcs* resistance gene in ‘Peking’ was found located within a 2.1-cM interval between markers AACCTA178 and Satt244 (Yang et al., 2001). A QTL, defined by RAPD marker CSOP1800C, CSOPA21250C, and CSOUB11100C were found to be associated with Brazil Race 4 (Sebastio et al., 2002). Though *Rcs3* and this QTL were located in the same chromosome, this QTL was non-allelic to *Rcs3*. These identified gene or QTL spanned fairly large genomic regions because of the relatively low density of molecular markers, which further limited their application in MAS efforts. In addition, presently molecular markers of resistance gene of few resistance sources were developed, and the genetic bases of more resistance source still need to be dissected.

Genome-wide association studies (GWASs), based on high-density SNP markers and the natural population with wider phenotypic variation, have more extensive recombination events and shorter LD block. Therefore, GWASs could significantly improve the resolution and accuracy of marker-phenotype associations than traditional linkage analysis. At present, GWAS has been well applied in dissecting genetic basis of resistance in soybean cyst nematode (Han et al., 2015; Zhang et al., 2016), white mold (Zhao et al., 2015; Boudhrioua et al., 2020), and phytophthora root or stem-rot (Schneider et al., 2016; Zhao et al., 2020). By early 2021, no studies have been conducted to identify QTL underlying the resistance to China race 1 of soybean FLS.

In this study, we collected a panel of 183 soybean accessions to evaluate their resistances to FLS race 1, and conducted a GWASs based on the association population and the 23,156 SNPs developed by the specific locus amplified fragment-sequencing (SLAF-seq). The objective was to screen resistant soybean germplasm adapted to the Northeast China, to identify QTNs with pronounced effects on FLS race 1 resistance, and to find potential functional genes near the peak SNPs.

## Materials and Methods

### Plant Materials and Resistance Evaluations

A total of 183 (landraces and elite cultivars) tested soybean samples, collected from China, were used to evaluate resistance to FLS, and the subsequent reduced-sequencing (Supplementary Table 1). The seeds of all the tested samples were surface sterilized, and then were germinated at growth room (28°C) until the radicle reached approximately 2 cm. Seedlings were planted in plastic cups filled with sterilized quartz sand under controlled environment (16 h day/8 h night cycle, 28°C and a relative humidity of 50%). An FLS China race 1 spore was suspended in a sterile aqueous solution containing 0.015% of the surfactant Tween 20 to a final concentration of 10^6^ spores ml^–1^. The trifoliate leaves of tested plant materials were challenged with FLS China race 1 spore at their V3–V4 growth stage. After 14 days, the disease severity was evaluated from 0 to 9 scale (Carmona et al., 2009). 0 and 9 denoted no disease and = 90% leaf tissue diseased. The disease index (DI) was then calculated to evaluate the resistance of each tested sample. The resistance identification of all accessions was repeated three times. In total ten plants were treated for each repetition. The tested sample without symptoms (DI = 0.00) was believed as resistant to FLS China race 1.

### Sequencing and Genotyping Data Collection

Genomic DNA was extracted from a bulk of fresh leaf tissue of each accession *via* the CTAB method, and then genotyped based on the specific locus amplified fragment-sequencing (SLAF-seq) methodology (Sun et al., 2013). The combination of the barcode method and Illumina Genome Analyzer II system (Illumina Inc., San Diego, CA, United States) was used to obtain 45-bp sequence reads at both ends of each library. The alignment between the raw paired-end reads and soybean reference genome Williams 82 (Version: Glyma.Wm82.a2) was performed *via* Short Oligonucleotide Alignment Program 2 (SOAP2) software. In SNP calling, the minor allele frequency (MAF) was set as 0.05. The genotype was defined as heterozygous when the depth of minor allele was larger than one-third of the total depth of the sample.

Based on the extreme phenotypic values for FLS resistance, 20 soybean lines (10 resistant lines with a lower level and ten susceptible lines with a higher level on disease index), were screened for a genome re-sequencing with 10-fold in depth on an Illumina HiSeq 2500 sequencer. Paired-end re-sequencing reads were mapped to the reference genome *via* BWA (Version: 0.6.1-r104) with the default parameters (Zhou et al., 2015). SAMtools48 (Version: 0.1.18) software was used in converting mapping results into the BAM format and filtering the unmapped and non-unique reads. Duplicated reads were filtered with the Picard package (Version: 1.87)^1^. The coverage of sequence alignments was computed *via* the BEDtools (Version: 2.17.0) coverage Bed program. SNP detection was conducted through the Genome Analysis Toolkit (GATK, version 2.4-7-g5e89f01) and the SAMtools. Only the SNPs identified by both methods could be subsequently analyzed, and the ones would be discarded whose allele frequencies were lower than 1% in the population. The annotations of SNPs were performed based on the reference genome using the package ANNOVAR (Version: 2013-08-23) (Zhou et al., 2015).

### Analysis of Population Structure and Linkage Disequilibrium

Population structure of the association panel was assessed through the principal component analysis (PCA) approach of GAPIT software (Lipka et al., 2012). LD between pairs of SNPs was determined based on the SNP threshold value (MAF > 0.05 and missing data <3%) and *r*^2^ (squared allele frequency correlations) by using the software TASSEL version 3.0 (Bradbury et al., 2007). Compared to GWAS, missing SNP genotypes were not imputed with the major allele prior to LD analysis. The parameters set in the program included MAF (>0.05) and the integrity of each SNP (>80%).

### Genome-Wide Association Mapping

Compressed mixed linear model (CMLM) in GAPIT was utilized to identify the association signals in relation to FLS race 1 resistance based on the 23,156 SNPs from 183 soybean accessions (Lipka et al., 2012). The significance threshold for the association between SNP and trait was determined by −log10(P) ≥ 3 (Yan et al., 2017; Sui et al., 2020).

### Prediction of Candidate Genes Controlling Resistance to China Race 1 of Frogeye Leaf Spot

Candidate genes located in the 200-kb genomic region of each peak SNP, were classified and annotated with the reference genome Williams 82^2^. Based on the data of the genomic re-sequencing, SNP variations (located in exonic regions, splicing sites, 5′ UTRs and 3′ UTRs, intronic regions, and upstream and downstream regions) were selected and used to identify FLS-related haplotypes using the General Linear Model (GLM) method in TASSEL version 3.0 (Bradbury et al., 2007). Significant SNPs related to the target trait were claimed when the test statistics reached *P* < 0.01 and *P* < 0.05. PlantCARE online software was used to predict the *cis-*acting elements in the promoter regions of the candidate genes^3^.

### Expression Patterns of Candidate Genes

Among the association panel, ‘HF55’ (resistant to race 1, carried all of beneficial alleles of SNPs associated FLS resistance) and ‘TD50’ (susceptible to race 1, without any beneficial alleles for FLS resistance) were used to investigate the expression patterns of the selected potential candidates by quantitative RT-PCR (qRT-PCR) analysis in order to ensure that the candidate gene transcription in response to FLS is directly related to the GWAS results. The leaves of each soybean accession from the FLS inoculation and the control group were collected at 0, 2, 4, 8, 12, 24, 36, 48, 60, and 72 h. The qRT−PCR assay was conducted on ABI 7500 Fast instrument by using SuperReal PreMix Plus (SYBR Green) Kit (TIANGEN, FP205). The *GmActin4* (GenBank accession no. AF049106) was used as the internal standard control of soybean. The sequences of the primer pairs used for amplifying the candidate genes were listed in Supplementary Table 2.

### Functional Verification of Candidate Genes Through Virus-Induced Gene Silencing Assay

‘HF55,’ a resistant soybean line to FLS race 1, harbored no special resistance to lentivirus (Zhang et al., 2015), thus, it was used to isolate candidate genes and to conduct the VIGS assay. The full-length cDNA sequences of the candidate genes were amplified using the specific-primers (Supplementary Table 2), and then cloned into the VIGS vector *pTRV2*. The *TRV1*, *TRV2-00* (negative control), *TRV2-PDS* (positive control), and the recombinant plasmids were transformed to *Agrobacterium strain* GV3101, respectively. The TRV-based VIGS assay were performed according to Liu’s protocol (Liu et al., 2002), when the first trifoliate soybean leaves of HF55 were fully expanded. About 2 weeks after the infiltration, qRT-PCR was used to measure the mRNA levels of the silenced genes among the serious infected leaves. All the infiltrated and control wild-type plants without infiltration were then inoculated FLS China race 1 to evaluate the infection condition. The VIGS experiments for each group were repeated three times with five plants at each time.

## Results

### Phenotypic Analysis of the Soybean Resistance to Frogeye Leaf Spot Race 1

**FIGURE 1:**
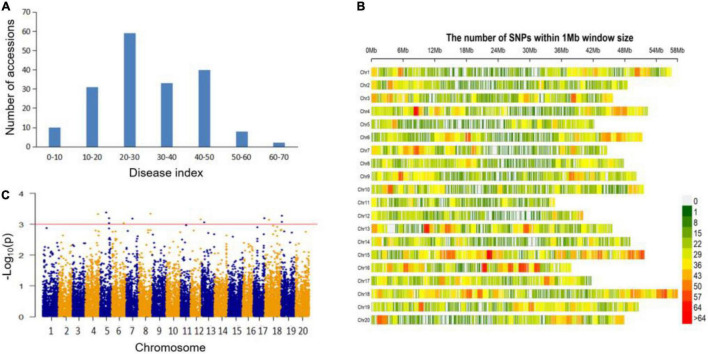
Genome wide association study on soybean resistance to FLS race 1. **(A)** Distribution of FLS race 1 resistance among 183 soybean accessions. **(B)** SNP density and distribution across the 20 soybean chromosomes. **(C)** Manhattan plot of association mapping of FLS race 1 resistance.

The disease index (DI) of 183 soybean germplasm were calculated 14 days after inoculating FLS China race 1 onto each tested plant (Supplementary Table 1). As was shown in Figure 1A and Table 1, the DI value ranged broadly, from the minimum of 0.00 to the maximum of 66.67. Three soybean accessions with the DI of 0.00, including Z30 from China, Domaka Tolisa-A from Yugoslavia and L-9 from United States, were believed as resistant to FLS China race 1 (Supplementary Table 1). The mean value, coefficient of variation (CV), skewness and kurtosis of the whole association panel were also shown in Table 1. The absolute values of kurtosis and skewness distributed nearly normal without saliency, indicating that the association panel was suitable for GWAS analysis.

**TABLE 1:** Statistical analysis and variation for FLS race 1 resistance among the association panel.

Trait	Min	Max	Mean	CV (%)	Skewness	Kurtosis
Disease index	0.00	66.67	30.96	42.5	0.08	−0.49

### Sequencing and Single-Nucleotide Polymorphism Distribution

All the 183 soybean accessions were sequenced on the basis of the extracted total DNA through the specific locus amplified fragment-sequencing (SLAF-seq) approach. For each soybean sample, an average of 49,571 high-quality tags were identified from 153 million paired end reads, with a read length of 45 bp and a sequencing depth of 6.51-fold. After deleting the markers with minor allele frequencies (MAFs) lower than 0.05, we finally obtained 23,156 high-quality SNP markers (MAF ≥ 0.05, missing data ≤0.03) distributed on 20 soybean chromosomes, with which we then performed a GWAS analysis (Figure 1B). These developed SNPs spanned approximately 947.01 Mbp and covered nearly 86.09% of the soybean genome. The number of SNPs among the 20 chromosomes varied from the least of 687 on chromosome (Chr.) 11 to the most of 1,733 on Chr.18, and the mean SNP per chromosome was about 1,158. The marker density on different chromosomes also varied greatly (from 33.44 kb per SNP on Chr.18 to 50.48 kb per SNP on Chr.11) with a genome-wide average of approximately one SNP for every 40.90 kb (Figure 1B).

### Genome-Wide Association Mapping of Soybean Resistance to Frogeye Leaf Spot China Race 1

To characterize the population structure, the average distance of LD decay was analyzed. The mean LD decay of the population was estimated as 189.2 kb when *r*^2^ dropped to about 0.3 (Supplementary Figure 1A). The group stratifications of the association panels were scanned through the principal component (PC) with the full set of 23,156 SNP markers. The results showed that the PC1, PC2, and PC3 could explain 12.91% of the genetic variations. The inflection point occurred at PC3, indicating that the first three PCs were main factors affecting the population structure (Supplementary Figures 1B,C). For further GWA-mapping, the kinship among accessions of the association panel was estimated. It revealed that a lower level of familial relationships was presented from the distribution of the pairwise relative kinship coefficients (Supplementary Figure 1D).

The marker-trait associations were conducted based on the phenotypic values and the complete set of high-quality SNPs. These were analyzed by the compressed mixed linear model (CMLM). Totally, 13 quantitative trait nucleotides (QTNs) associated with soybean FLS race 1 resistance were detected (Figure 1C and Table 2). Among them, two peak SNPs, Gm19:714816 and Gm19:839834 (at the position of 714816 bp and 839834 bp on Chr.19) were identified close to each other that could contain many overlapped candidate genes. Similarly, Gm05:31332397 and Gm05:31499885 (at the position of 31332397 and 31499885 on Chr.05) might also be the major loci. Compared with the previous studies, we found all the 13 QTNs were the novel loci for the FLS race 1 resistance. To further verify whether these association signals were related to the target trait, the allelic effects were analyzed (Table 2). The results presented that the accessions carrying the beneficial allele (allele 1) showed lower decease index compared with those with the susceptible allele (allele 2), demonstrating that different alleles for each QTN could have significant effects on FLS race 1 resistance. These QTNs and the beneficial alleles (allele 1) were of the great value in MAS for soybeans with higher resistance to FLS race 1.

**TABLE 2:** Peak SNPs and beneficial alleles associated with FLS race 1 resistance identified by GWAS.

SNP	Chr.	Position	−log10(P)	MAF	Allele 1/Allele 2	Average disease index of accessions with allele 1	Average disease index of accessions with allele 2	Average disease index of the population
Gm04:45376050	4	45376050	3.33	0.11	A/C	26.42	31.47	30.96
Gm05:22226540	5	22226540	3.37	0.12	C/T	29.56	41.67	30.96
Gm05:31332397	5	31332397	3.03	0.08	T/G	21.43	31.71	30.96
Gm05:31499885	5	31499885	3.19	0.05	A/G	21.17	31.48	30.96
Gm06:42106014	6	42106014	3.04	0.12	C/A	29.87	37.85	30.96
Gm07:20934425	7	20934425	3.17	0.05	T/A	30.06	39.90	30.96
Gm08:41958637	8	41958637	3.34	0.16	T/C	30.16	35.12	30.96
Gm12:37109252	12	37109252	3.15	0.08	A/G	30.23	38.12	30.96
Gm13:9742355	13	9742355	3.06	0.48	A/G	28.04	34.03	30.96
Gm17:38293751	17	38293751	3.20	0.09	G/A	30.20	37.94	30.96
Gm18:14428467	18	14428467	3.14	0.08	A/C	23.73	31.85	30.96
Gm19:714816	19	714816	3.07	0.07	A/T	30.66	34.58	30.96
Gm19:839834	19	839834	3.27	0.07	G/A	30.69	33.97	30.96

### Prediction of the Potential Genes Conferring Resistance to Frogeye Leaf Spot

A total of 119 potential genes were screened in the 200-kb flanking region of the 13 detected QTNs (Supplementary Table 3). Except for two genes with unknown function or uncharacterized protein domain, the remaining 117 genes were categorized into diverse functional groups that usually participated in various biological processes based on the Gene Ontology database^4^. Among these processes, most genes were involved in the RNA regulation of transcription, protein synthesis/modification/degradation, hormone metabolism, secondary metabolism, signaling, stress, and transport (Supplementary Figure 2). According to existed reports, some of these candidate genes have been supposed to be important in plant disease-related response.

*Glyma.17G228300* is a member of alpha/beta-hydrolases superfamily protein, which were the receptors in mediating signaling mechanisms involved in the regulation of parasitic plants or fungi (Lumba et al., 2017; Nasir et al., 2020). Zhou et al. (2016) reported that *GRMZM2G031169* [a maize NAD(P)-binding Rossmann-fold superfamily protein] was responsive to maize rough dwarf disease. In this study, we found two genes, *Glyma.19G006900* and *Glyma.19G007200* (located near QTN Gm19:714816 of Chr.19) contained the same functional protein family, which might aid in soybean FLS resistance. Kumar et al. (2016) indicated that *AdZADH2* (a novel zinc-binding alcohol dehydrogenase 2 from *Arachis diogoi*) conferred resistance to tomato late leaf spot pathogen, which harbored the same domain with *Glyma.19G008600* and *Glyma.19G008700* (located near QTN Gm19:714816 of Chr.19). *Glyma.19G008900* encoded a prenyltransferase protein that contributed to plant disease resistance (Bonhoff et al., 1986; Sukumaran et al., 2018). In addition, *Glyma.08G301200*, *Glyma.08G301800*, and *Glyma.12G212500* (located near QTNs Gm08:41958637 and Gm12:37109252, respectively) contained the common disease-resistant proteins that were deemed to be important regulators in resisting plant diseases (Yang et al., 2010; Chandra et al., 2017).

### Gene-Based Association Analysis of Potential Genes for Soybean Resistance to Frogeye Leaf Spot

**FIGURE 2:**
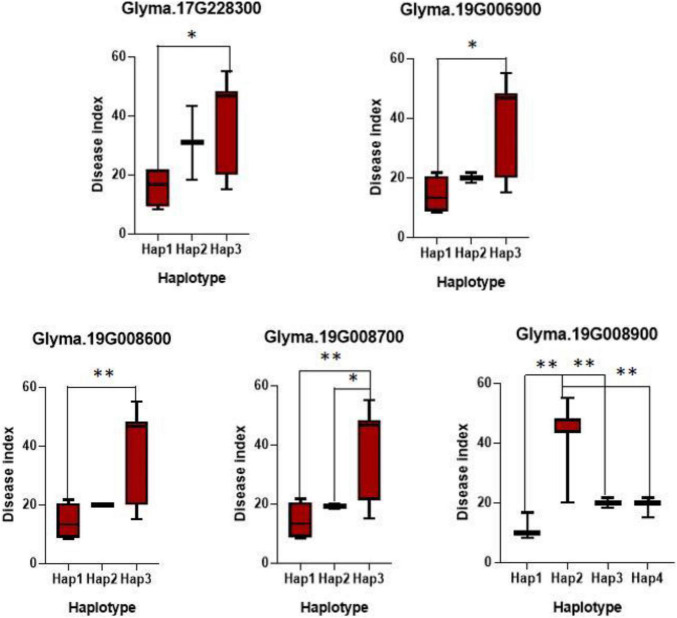
Haplotypes analysis of genes with variations related with FLS race 1 resistance. The ^∗^ and ^∗∗^ suggested significance of ANOVA at *p* < 0.05 and *p* < 0.01, respectively.

To further confirm the key variations of the potential genes for soybean resistance to FLS China race 1, a gene-based association analysis for all the detected genes was conducted. Totally, 1,520 SNPs from 117 candidates were identified among 20 soybean accessions (10 resistant and 10 susceptible lines) on basis of genome re-sequencing (MAF > 0.1). GLM method in TASSEL was used for the gene-based association analysis, and the significant SNPs (only SNPs with *P*-value of 0.01) were used to identify positive SNPs. Finally, 17 SNPs from seven genes were significantly associated with soybean resistance to FLS China race 1 (Table 3). These SNPs mostly located in intronic, upstream, downstream, UTR3, and UTR5 regions. In addition, the effects of different alleles from each peak SNP of candidate genes were analyzed. Of the seven genes with SNPs significantly related to FLS resistance, the disease indexes of soybean accessions with beneficial haplotypes of five genes were significantly lower than soybean accessions with other haplotypes (Figure 2). These beneficial alleles from the potential genes would be helpful in breeding soybean cultivars with resistance to FLS.

**TABLE 3:** Gene-based association analysis of candidate genes.

Gene ID	Chromosome	SNP position (bp)	Region	Alleles	−log10(P)	Functional annotation
Glyma.17G228300	17	38316228	Upstream	C/T	2.14	Alpha/beta-Hydrolases superfamily protein
		38318984	Intronic	T/C	2.16	
		38325101	UTR3	C/T	2.19	
Glyma.17G228700	17	38363818	Upstream	T/G	2.04	Cytokinin-responsive GATA factor 1
Glyma.19G006900	19	692367	UTR5	T/G	2.12	NAD(P)-binding Rossmann-fold superfamily protein
		692384	UTR5	A/C	2.99	
Glyma.19G008600	19	843604	Intronic	A/G	2.12	Oxidoreductase, zinc-binding dehydrogenase family protein
		844761	Downstream	A/G	2.50	
Glyma.19G008700	19	846949	Downstream	G/A	2.88	Oxidoreductase, zinc-binding dehydrogenase family protein
		846981	Downstream	G/A	2.12	
Glyma.19G008900	19	857132	Downstream	G/A	3.80	Prenyltransferase family protein
		857166	Downstream	T/C	2.61	
		857218	Downstream	A/G	2.12	
		858535	UTR3	A/G	2.15	
		858937	Intronic	G/A	2.65	
Glyma.19G009000	19	865198	UTR3	G/C	2.21	Formate dehydrogenase

### Expression Pattern Analysis of Potential Genes for Soybean Resistance to Frogeye Leaf Spot

**FIGURE 3:**
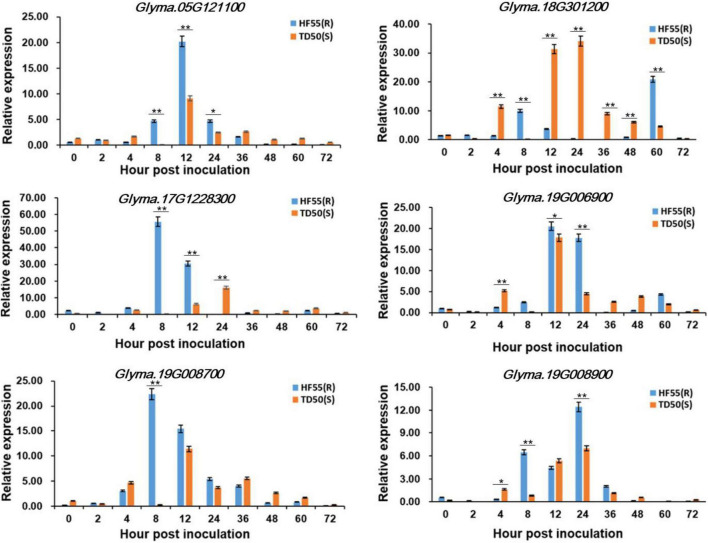
Expression patterns of the candidate genes. The mRNA levels of each candidate gene were analyzed in resistant soybean accession ‘HF55’ and susceptible soybean accession ‘TD50.’ The values were the mean ± SD of three biological replicates. ** and * represented the significance at *p* < 0.01 and *p* < 0.05, respectively.

To predict and investigate whether the candidate genes respond to the stress of FLS, the 2.0-kb upstream sequences of the genes were selected for *cis-*acting elements analysis. We finally screened six genes that contained the elements involved in defense and stress-related responses, and analyzed their expression patterns among ‘HF55’ (FLS resistant soybean line) and ‘TD50’ (FLS susceptible soybean line) based on qRT-PCR (Figure 3 and Supplementary Table 4). Of them, *Glyma.08G301200*, which encoded a TIR-NBS-LRR protein, exhibited significant down-regulated expression by FLS race 1 in the susceptible line ‘TD50’ and reached the peak at 24 h after inoculation; however, in the resistant line ‘HF55,’ the expression had no salient change during the early period until a sharp increase at the 60 h after inoculation. The other five genes, including *Glyma.05G121100*, *Glyma.17G228300*, *Glyma.19G006900*, *Glyma.19G008700*, and *Glyma.19G008900*, presented a prominent upregulation in the resistant soybean line and reached a peak at 8, 12, or 24 h after inoculation, respectively.

### Silencing Candidate Genes in Soybean Showed the Increased Susceptibility to Frogeye Leaf Spot

**FIGURE 4:**
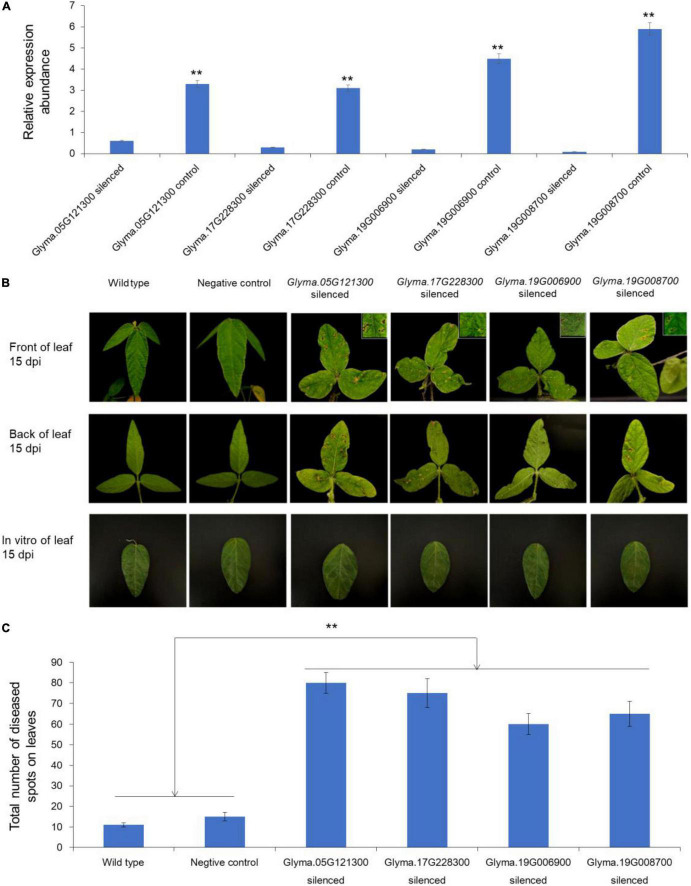
Functional verification of candidate genes through VIGS assay. **(A)** Relative expression abundance of each candidate genes associated with FLS resistance in gene silenced plants and wild type plants. **(B)** Symptoms on the leaves of gene silenced plants and wild type plants after inoculation by *Cercospora sojina Hara*. **(C)** Total number of diseased spots on leaves of gene silenced and control plants. ** represented the significance at *p* < 0.01.

To understand the functions of potential genes, the TRV*-*based VIGS system were performed. According to the results of haplotype analysis and expression pattern analysis, we finally selected four genes (*Glyma.05G121100*, 627 bp; *Glyma.17G228300*, 846 bp; *Glyma.19G006900*, 1,089 bp; and *Glyma.19G008700*, 969 bp) to verify their functions in soybean FLS resistance (Supplementary Figure 3). After 2 weeks of the inoculation, the expression levels of silenced candidate genes were detected. The results presented that the abundances of the four genes in leaves were significantly lower than that in blank control, which indicated that the expression of these genes were effectively suppressed (Figure 4A). Subsequently, the gene silenced soybean plants and wild type soybean plants were inoculated by FLS race 1 *via* both *in vivo* and *in vitro* methods. About 15 days after inoculation, the symptoms of FLS in the leaves of candidate genes silenced plants were obvious, but the wild type plants were with few symptoms. As was shown in Figure 4B, the disease spots were obvious on the front of leaves from the living plants in which the candidate genes were silenced. The symptoms on the back of the above leaves were more pronounced. The number of diseased spots on leaves of candidate gene silenced plants was significantly more than that of control plants (Figure 4C). The results demonstrated that the silencing of *Glyma.05G121100*, *Glyma.17G228300*, *Glyma.19G006900*, and *Glyma.19G008700* made the resistant cultivar ‘HF55’ sensitive to FLS race 1. Therefore, we believed that these four genes could play important roles in resisting soybean FLS race 1.

## Discussion

Soybean FLS has been a common fungal disease in most soybean-growing countries throughout the world (Ding et al., 2012; Mengistu et al., 2014; Soares et al., 2015; Gu et al., 2020). In this study, the disease index of Z30, Domaka Tolisa-A and L-9 were 0.00, exhibiting a higher resistance level to China race 1, which could be utilized as resistance sources to breed new resistance cultivars in China.

For the past two decades, many researchers were devoted to identifying DNA markers that were associated with the resistance to FLS for MAS (Mian et al., 2008). Polymorphic RAPD, AFLP, and SSR markers were used to map the *Rcs3* gene within different soybean genetic populations (Mian et al., 1999; Yang et al., 2001). More recently, Pham et al. (2015) narrowed down the *Rcs* (PI 594891) resistance region to a 72.6 kb and *Rcs* (PI 594774) region to a 540 kb based the cross populations between the cultivar ‘Blackhawk’ and the accessions PI 59489 and PI 594774 with 91 SNP markers. Based on these efforts, *Rcs3* gene has been applied gradually in soybean production, however, very few studies on molecular markers have been reported on *Rcs1* for the resistance to China race 1. To date, only Dong et al. (1999) reported two RAPD markers (OPK03840 and OPM171700) associated with resistance QTL of China race 1, however, this resistance QTL could not be identified on the specific genomic regions for character of RAPD markers. Thus, these two RAPD markers were not effectively utilized in MAS. Based on the diverse samples, GWAS offered an effective strategy to fine map FLS resistance QTL for a large number of historical recombination events and high-density SNP markers that lead to the rapid decay of LD (Han et al., 2015). In this study, all 13 association QTN were the novel ones, and of them, Gm05:31332397 and Gm05:31499885, Gm19:714816, and Gm19:839834 were close genomic region each other. In addition, these samples with the “beneficial” allele from these identified QTNs had higher resistance than those of others with the “inferior” allele. These QTNs and the beneficial alleles may be valuable in MAS for variety with FLS race 1 resistance.

To date, no specific gene for FLS resistance were reported. Missaoui et al. (2007) predicted two candidate genes including ATP-binding cassette (ABC) transporter and leucine-rich repeat (LRR) sequence based on screening of bacterial artificial chromosome (BAC) end sequences near two simple sequence repeat markers of *Rcs3* gene. Among them, LRR gene, recognize and transmit pathogen-derived signals, had no nucleotide variations between resistance and susceptible accessions. Thus, these two-candidate gene still were not verified to resistance to FLS. In this study, a total of seven genes in the 200-kbp flanking region of these identified QTNs were verified associated with resistance to FLS China race 1 through the gene-based association and haplotype analysis (Table 3). In addition, the expression patterns of 6 genes, containing *cis-*elements related to defense and stress, were also analyzed (Supplementary Table 4). The results showed the expression level of *Glyma.05G121100*, *Glyma.17G228300*, *Glyma.19G006900*, *Glyma.19G008700*, and *Glyma.19G008900* were significantly up-regulated after inoculation in the resistant accession in comparison with that in the susceptible accession. Their expression level peaked at the time of 8, 12, and 24 h, which suggested that these candidates could be involved in the response to FLS race 1 resistance. Among them, resistance of 4 candidate genes were further verified based on VIGS experiments. Of these 4 genes, *Glyma.05G121100* belonged to RNA-binding (RRM/RBD/RNP motifs) family protein with retrovirus zinc finger-like domain; *Glyma.17G228300* was an alpha/beta-Hydrolases superfamily protein that participated the signaling mechanisms involved in the management of parasitic plants or fungi (Lumba et al., 2017; Nasir et al., 2020); *Glyma.19G006900* encoded a NAD(P)-binding Rossmann-fold protein that could be responsive to plant disease (Zhou et al., 2016); *Glyma.19G008700* was a member of zinc-binding dehydrogenase family protein, which harbored the same domain with *AdZADH2*, whose important role in tomato resistance to late leaf spot has been reported by Kumar et al. (2016). As expected, the disease symptoms in leaves carrying the silenced candidate genes were more obvious than that in blank control. Thus, these four genes played an important role in soybean FLS race 1 resistance, and their clear functions and specific mechanisms deserve further analysis.

## Data Availability Statement

The original contributions presented in the study are included in the article/Supplementary Material, further inquiries can be directed to the corresponding author/s.

## Author Contributions

YH and XZ designed and supervised the research. MS and CN conducted the experiment. YJ, ZC, and NL evaluated phenotypic values. YZ, YL, and WT analyzed the data. YJ, XZ, YH, and WL wrote the manuscript. All authors read and approved the manuscript.

## Conflict of Interest

The authors declare that the research was conducted in the absence of any commercial or financial relationships that could be construed as a potential conflict of interest.

## Publisher’s Note

All claims expressed in this article are solely those of the authors and do not necessarily represent those of their affiliated organizations, or those of the publisher, the editors and the reviewers. Any product that may be evaluated in this article, or claim that may be made by its manufacturer, is not guaranteed or endorsed by the publisher.

## Footnotes

1
picard.sourceforge.net

2
https://phytozome.jgi.doe.gov/pz/portal.html

3
http://bioinformatics.psb.ugent.be/webtools/plantcare/html/

4
http://geneontology.org/
