# Supplementary material for: Genome-Wide Association Analysis and Gene Mining of Resistance to China Race 1 of Frogeye Leaf Spot in Soybean
Source: Front Plant Sci. 2022 Jun 22;13:867713. doi: 10.3389/fpls.2022.867713 (PMC9257224; doi:10.3389/fpls.2022.867713)
Supplement: Supplementary file 1 [file Data_Sheet_1.docx]

**Supplementary material**

**
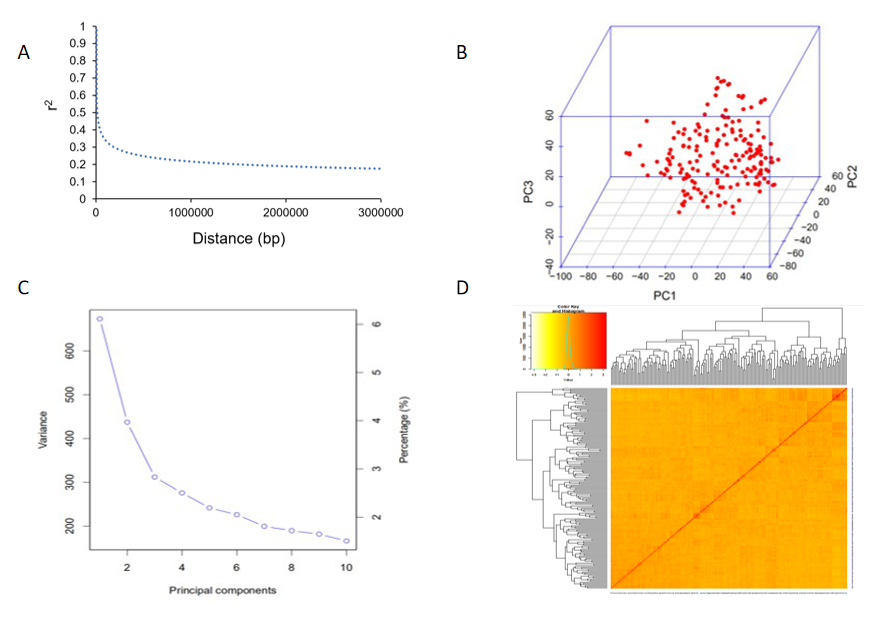
**

**Figure S1. Genetic feature of mapping population**. (A) The linkage disequilibrium (LD) decay of genome-wide association study (GWAS) population. (B) The first three principal components of the more than 20,000 SNPs used in the GWAS. (C) Population structure of soybean germplasm collections as reflected by principal components. (D) A heatmap of the kinship matrix among the 183 soybean accessions.


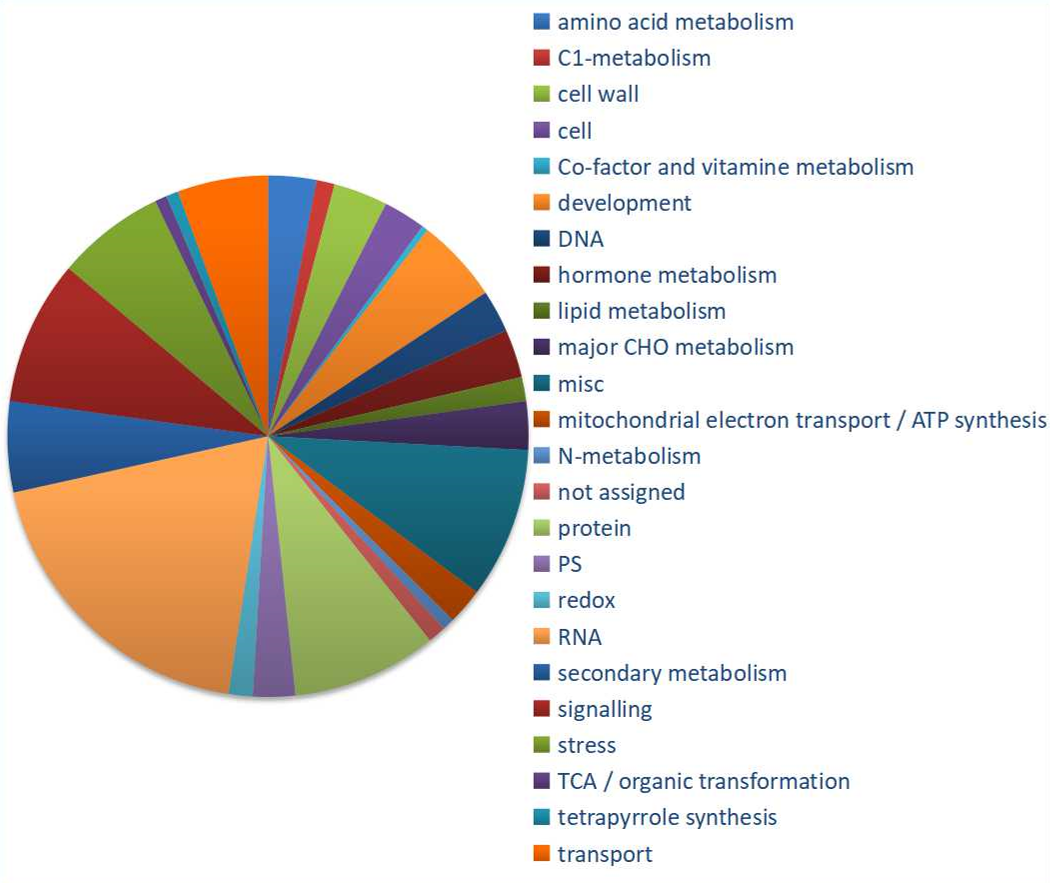


**Figure S2.** Functional classifications of the candidate genes for soybean FLS race 1 resistance.


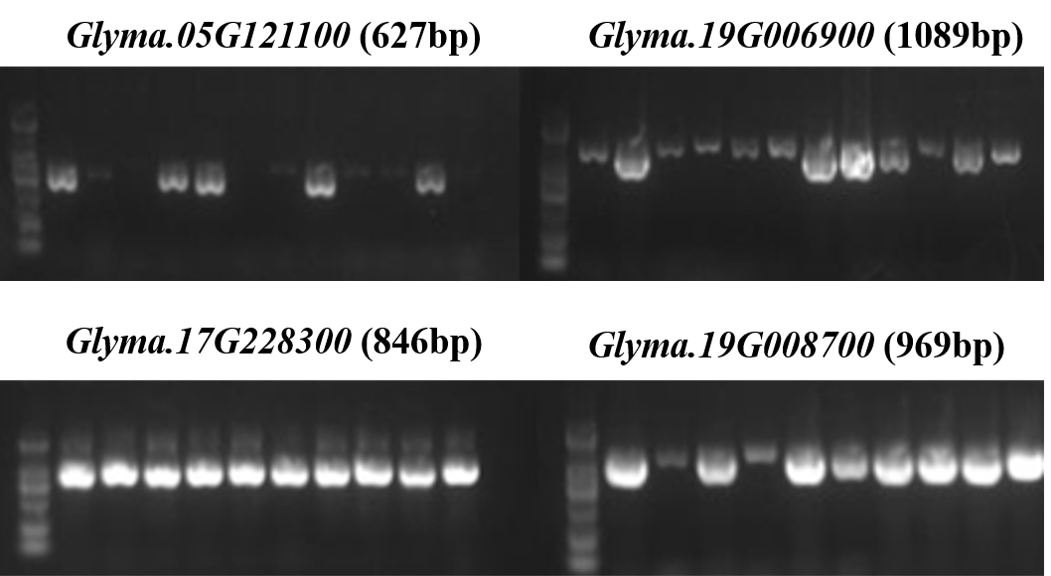


**Figure S3.** PCR verification of the VIGS vector construction.

**Table S1.** List of 183 soybean accessions with phenotype values.

| Name | Country | Latitude (°N) | Longitude (°W) | Average of disease index |
| --- | --- | --- | --- | --- |
| 4five-star | China | 39.19 | 116.29 | 48.33 |
| 4yellow | China | 48.29 | 128.08 | 46.67 |
| Aika166 | Rumania | 44.23 | 26.10 | 33.33 |
| amsoy | USA | 41.83 | 92.91 | 21.67 |
| B1361 | China | 48.29 | 128.08 | 21.67 |
| B1484 | China | 48.29 | 128.08 | 43.33 |
| B1873 | China | 48.29 | 128.08 | 38.33 |
| B4834 | China | 48.29 | 128.08 | 53.33 |
| BD14 | China | 48.29 | 128.08 | 26.67 |
| BD16 | China | 48.29 | 128.08 | 66.67 |
| BF9 | China | 48.29 | 128.08 | 46.67 |
| Big black | China | 36.23 | 118.31 | 33.33 |
| Bmoshi | China | 43.35 | 126.29 | 6.67 |
| Boige du | Germany | 52.31 | 13.20 | 21.67 |
| Cateye | China | 37.34 | 100.30 | 28.33 |
| Cdou1 | China | 37.34 | 84.79 | 30.00 |
| Chamoshidou | China | 43.35 | 126.29 | 20.00 |
| charleston | USA | 41.83 | 92.91 | 41.67 |
| Conrad | USA | 41.83 | 92.91 | 18.33 |
| Cse | China | 43.35 | 126.29 | 28.33 |
| Datenhei | China | 39.19 | 116.29 | 43.33 |
| DN L-13 | Canada | 43.40 | 79.25 | 33.33 |
| DN07-909 | China | 48.29 | 128.08 | 25.00 |
| DN1068 | China | 48.29 | 128.08 | 30.00 |
| DN42 | China | 48.29 | 128.08 | 51.67 |
| DN43 | China | 48.29 | 128.08 | 11.67 |
| DN44 | China | 48.29 | 128.08 | 45.00 |
| DN46 | China | 48.29 | 128.08 | 16.67 |
| DN47 | China | 48.29 | 128.08 | 23.33 |
| DN48 | China | 48.29 | 128.08 | 43.33 |
| DN49 | China | 48.29 | 128.08 | 30.00 |
| DN50 | Canada | 43.40 | 79.25 | 48.33 |
| DN56 | China | 48.29 | 128.08 | 21.67 |
| DN594 | China | 48.29 | 128.08 | 38.33 |
| DN93046 | China | 48.29 | 128.08 | 56.67 |
| Domaka Tolisa-A | Yugoslavia | 46.30 | 14.30 | 0.00 |
| Dongnong8004 | China | 48.29 | 128.08 | 20.00 |
| Douludou | China | 43.35 | 126.29 | 30.00 |
| Dunajika | Russia | 61.50 | 98.00 | 40.00 |
| DY2004-5 | USA | 41.83 | 92.91 | 15.00 |
| Early18 | China | 40.15 | 116.28 | 3.33 |
| Fendiyellow | China | 43.35 | 126.29 | 25.00 |
| Fenshou6 | China | 48.29 | 128.08 | 13.33 |
| FZmoshidou | China | 48.29 | 128.08 | 35.00 |
| Gonye04-141 | China | 43.35 | 126.29 | 26.67 |
| H04-1824 | China | 48.29 | 128.08 | 61.67 |
| H05-31 | China | 48.29 | 128.08 | 21.67 |
| H05-991 | China | 48.29 | 128.08 | 18.33 |
| Hangbaodou | China | 40.85 | 122.00 | 41.67 |
| Harosoy | USA | 41.83 | 92.91 | 18.33 |
| Harvest10 | China | 48.29 | 128.08 | 20.00 |
| HD5 | China | 39.19 | 116.29 | 13.33 |
| Heersong2 | Ukraine | 50.28 | 30.29 | 6.67 |
| Heilongjiang41 | China | 48.29 | 128.08 | 18.33 |
| Heimodou | China | 43.35 | 126.29 | 40.00 |
| Heinong33 | China | 48.29 | 128.08 | 23.33 |
| Heinong37 | China | 48.29 | 128.08 | 36.67 |
| Heinong48 | China | 48.29 | 128.08 | 45.00 |
| HF11 | China | 48.29 | 128.08 | 15.00 |
| HF25 | China | 48.29 | 128.08 | 55.00 |
| HF29 | China | 48.29 | 128.08 | 41.67 |
| HF35 | China | 48.29 | 128.08 | 48.33 |
| HF37 | China | 48.29 | 128.08 | 23.33 |
| HF45 | China | 48.29 | 128.08 | 20.00 |
| HF47 | China | 48.29 | 128.08 | 48.33 |
| HF50 | China | 48.29 | 128.08 | 43.33 |
| HF52 | China | 48.29 | 128.08 | 46.67 |
| HF55 | China | 48.29 | 128.08 | 10.00 |
| HH18 | China | 48.29 | 128.08 | 46.67 |
| HH38 | China | 48.29 | 128.08 | 40.00 |
| HH45 | China | 48.29 | 128.08 | 41.67 |
| HH48 | China | 48.29 | 128.08 | 36.67 |
| HHX | China | 48.29 | 128.08 | 46.67 |
| HJ03-286 | China | 44.49 | 111.70 | 31.67 |
| HJ04-528 | China | 44.49 | 111.70 | 25.00 |
| HN44 | China | 48.29 | 128.08 | 26.67 |
| HN55 | China | 48.29 | 128.08 | 26.67 |
| Huajian2 | China | 48.29 | 128.08 | 41.67 |
| HuaJiang4403 | China | 48.29 | 128.08 | 43.33 |
| HuJiao423 | China | 44.49 | 111.70 | 30.00 |
| HY446 | China | 39.19 | 116.29 | 26.67 |
| HYoutai | China | 43.35 | 126.29 | 25.00 |
| J06B7 | China | 39.19 | 116.29 | 43.33 |
| J100 | China | 43.35 | 126.29 | 23.33 |
| Jcha | China | 43.35 | 126.29 | 40.00 |
| JD12 | China | 39.19 | 116.29 | 25.00 |
| JD17 | China | 39.19 | 116.29 | 31.67 |
| JD33 | China | 40.85 | 122.00 | 10.00 |
| JD9 | China | 39.19 | 116.29 | 21.67 |
| Jin13 | China | 39.19 | 116.29 | 48.33 |
| Jishanpu | China | 48.29 | 128.08 | 25.00 |
| Jiyu89 | China | 43.35 | 126.29 | 50.00 |
| Jiyu94 | China | 43.35 | 126.29 | 23.33 |
| JL30 | China | 43.35 | 126.29 | 45.00 |
| JL47 | China | 43.35 | 126.29 | 28.33 |
| JN20 | China | 43.35 | 126.29 | 21.67 |
| JN21 | China | 43.35 | 126.29 | 31.67 |
| Jshanmoshi | China | 43.35 | 126.29 | 28.33 |
| JZ4-1 | China | 40.85 | 122.00 | 13.33 |
| K04-8579 | China | 48.29 | 128.08 | 48.33 |
| K29 | China | 48.29 | 128.08 | 56.67 |
| K30 | China | 48.29 | 128.08 | 11.67 |
| KB1 | China | 48.29 | 128.08 | 30.00 |
| Ken14 | China | 48.29 | 128.08 | 33.33 |
| Ken15 | China | 48.29 | 128.08 | 45.00 |
| Ken18 | China | 48.29 | 128.08 | 16.67 |
| Ken22 | China | 48.29 | 128.08 | 26.67 |
| KJ23 | China | 48.29 | 128.08 | 40.00 |
| KX2 | China | 40.15 | 116.28 | 25.00 |
| KX3 | China | 40.15 | 116.28 | 31.67 |
| KX3 | China | 40.15 | 116.28 | 48.33 |
| L-10 | USA | 41.83 | 92.91 | 8.33 |
| L-21 | USA | 41.83 | 92.91 | 23.33 |
| L-28 | Canada | 43.40 | 79.25 | 48.33 |
| L-5 | Canada | 43.40 | 79.25 | 16.67 |
| L-57 | USA | 41.83 | 92.91 | 25.00 |
| L-58Keburi | USA | 41.83 | 92.91 | 23.33 |
| L-59Peking | USA | 41.83 | 92.91 | 20.00 |
| L-79 | Canada | 43.40 | 79.25 | 21.67 |
| L-9 | USA | 41.83 | 92.91 | 0.00 |
| L98072 | China | 40.85 | 122.00 | 26.67 |
| LD23 | China | 40.85 | 122.00 | 28.33 |
| LD3 | China | 40.85 | 122.00 | 26.67 |
| LN2 | China | 40.85 | 122.00 | 23.33 |
| Lonqan | China | 48.29 | 128.08 | 53.33 |
| LP03-311 | China | 48.29 | 128.08 | 53.33 |
| LX1 | China | 40.85 | 122.00 | 25.00 |
| LX1 | China | 40.85 | 122.00 | 28.33 |
| MD14 | China | 44.49 | 111.70 | 16.67 |
| MD21 | China | 44.49 | 111.70 | 40.00 |
| MD9 | China | 44.49 | 111.70 | 15.00 |
| MF1 | China | 48.29 | 128.08 | 13.33 |
| NF11 | China | 48.29 | 128.08 | 33.33 |
| NF15 | China | 48.29 | 128.08 | 23.33 |
| Nova | Italy | 41.54 | 12.30 | 43.33 |
| Qian black | China | 48.29 | 128.08 | 23.33 |
| S02-339 | China | 48.29 | 128.08 | 45.00 |
| S03-3046 | China | 48.29 | 128.08 | 53.33 |
| S03-3952 | China | 48.29 | 128.08 | 28.33 |
| S04-5804 | China | 48.29 | 128.08 | 40.00 |
| S04-6018 | China | 48.29 | 128.08 | 20.00 |
| S05-7304 | China | 48.29 | 128.08 | 20.00 |
| S28 | China | 48.29 | 128.08 | 20.00 |
| S29 | China | 48.29 | 128.08 | 21.67 |
| S30 | China | 48.29 | 128.08 | 31.67 |
| Sixty days | China | 40.85 | 122.00 | 48.33 |
| Small Moshi | China | 48.29 | 128.08 | 33.33 |
| SN1 | China | 48.29 | 128.08 | 18.33 |
| SN10 | China | 48.29 | 128.08 | 26.67 |
| SN25 | China | 48.29 | 128.08 | 30.00 |
| SN8 | China | 48.29 | 128.08 | 28.33 |
| Stone | China | 39.19 | 116.29 | 25.00 |
| Sui20 | China | 48.29 | 128.08 | 23.33 |
| Suinong14 | China | 48.29 | 128.08 | 41.67 |
| Suinong4 | China | 48.29 | 128.08 | 30.00 |
| TD50 | China | 40.85 | 122.00 | 38.33 |
| TD51 | China | 40.85 | 122.00 | 40.00 |
| TD52 | China | 40.85 | 122.00 | 16.67 |
| TD54 | China | 40.85 | 122.00 | 46.67 |
| TD58 | China | 40.85 | 122.00 | 35.00 |
| Tejia | China | 40.85 | 122.00 | 36.67 |
| Tejia | China | 40.85 | 122.00 | 46.67 |
| TF8 | China | 40.85 | 122.00 | 21.67 |
| Tiefeng31 | China | 40.85 | 122.00 | 16.67 |
| TN13 | China | 43.35 | 126.29 | 18.33 |
| V111-4 | China | 43.35 | 126.29 | 10.00 |
| williams82 | USA | 41.83 | 92.91 | 38.33 |
| XB | China | 43.35 | 126.29 | 36.67 |
| Yanbojin | China | 48.29 | 128.08 | 28.33 |
| Yapiche | China | 48.29 | 128.08 | 20.00 |
| Z03-5179 | China | 40.15 | 116.28 | 46.67 |
| Z03-5334 | China | 40.15 | 116.28 | 41.67 |
| Z03-5373 | China | 40.15 | 116.28 | 48.33 |
| Z10 | China | 40.15 | 116.28 | 25.00 |
| Z20 | China | 40.15 | 116.28 | 31.67 |
| Z30 | China | 40.15 | 116.28 | 0.00 |
| Z6 | China | 40.15 | 116.28 | 45.00 |
| Z95-5388 | China | 40.15 | 116.28 | 40.00 |
| ZD27 | China | 40.15 | 116.28 | 48.33 |
| ZD35 | China | 40.15 | 116.28 | 15.00 |
| Zhonghuang35 | China | 40.15 | 116.28 | 26.67 |
| ZZ00-683 | China | 40.15 | 116.28 | 38.33 |
| ZZJ4133 | China | 40.15 | 116.28 | 33.33 |

**Table S2.** List of primers for gene amplification.

| Gene ID | Forward primer | Reverse primer |
| --- | --- | --- |
| GmACTIN | GATCTACCATGTTCCCAAGT | ATAGAGCCACCAATCCAGAC |
| qGlyma.05G121100 | CTGGTCATTTTGCTAGGGAATG | CTCATATGGTCCTGAACGATCA |
| qGlyma.08G301200 | CGGATAAGCATTTGTTACGTGT | ATTGGAATAAAGTACTGCACGC |
| qGlyma.17G228300 | CATCTTGCCTGCCTATTGATTC | CCAAAACTAAGCTGGTCAACTC |
| qGlyma.19G006900 | GAACCCACAAGTTTGTTTCCTC | ACCAAAGTAGGAAACTCCACAA |
| qGlyma.19G008700 | GCATTTGCACACTTGAAGACTA | GATATTCACGTTTGATCACGCA |
| qGlyma.19G008900 | ATGAGTGAAGAGAGCGAGAAAG | TTGGTAAATCTGAAACACCTGC |
| qGm-PDS | ATGGCCGCTTGTGGCTATATAT | GTTCAAGGGTTTTAGAGGACG |
| VIGS-Glyma.05G121100 | AGGTTACCGAATTCTATGTCTGACGTGGAAGAGTT | ATGGAGGCCTTCTAGTCAACGAAAGCCTCCCGAT |
| VIGS-Glyma.17G228300 | AGGTTACCGAATTCTATGGCCGATTGTGGTGCTGAAAG | ATGGAGGCCTTCTAGCTAGAATCGGGAATGAATAG |
| VIGS-Glyma.19G006900 | AGGTTACCGAATTCTATGGGTGTTGTACGGACGTGG | ATGGAGGCCTTCTAGCTAATATTCACTGTAACAC |
| VIGS-Glyma.19G008700 | AGGTTACCGAATTCTATGGCAAGCACTCCTAGCATT | ATGGAGGCCTTCTAGTTATGGAATGGGATGTAT |

**Table S3.** Candidate genes in the 200 kb flanking region of peak SNP.

| Peak SNP | Chromosome | Physical position (bp) | Gene | Distance to SNP(kb) | Functional annotation |
| --- | --- | --- | --- | --- | --- |
| Gm04:45376050 | 4 | 45376050 | Glyma.04G184000 | 86.13 | HCO3- transporter family |
|  |  |  | Glyma.04G184200 | 40.92 | myb domain protein 98 |
|  |  |  | Glyma.04G184300 | 21.68 | Glucose-6-phosphate/phosphate translocator-related |
|  |  |  | Glyma.04G184400 | 5.97 | SKP1/ASK-interacting protein 16 |
|  |  |  | Glyma.04G184600 | 31.85 | Phox (PX) domain-containing protein |
|  |  |  | Glyma.04G184800 | 5.31 | 12-oxophytodienoate reductase 1 |
|  |  |  | Glyma.04G184900 | 62.28 | tryptophan biosynthesis 1 |
| Gm05:22226540 | 5 | 22226540 | Glyma.05G091600 | 49.29 | 2-oxoglutarate (2OG) and Fe(II)-dependent oxygenase superfamily protein |
|  |  |  | Glyma.05G091900 | 0.18 | Ribosomal L5P family protein |
|  |  |  | Glyma.05G092000 | 0.05 | Di-haem cytochrome, transmembrane;Cytochrome b/b6, C-terminal |
|  |  |  | Glyma.05G092100 | 2.88 | photosystem II reaction center protein H |
|  |  |  | Glyma.05G092300 | 28.05 | ATP synthase subunit 1 |
|  |  |  | Glyma.05G092400 | 74.98 | HXXXD-type acyl-transferase family protein |
| Gm05:31332397 | 5 | 31332397 | Glyma.05G119500 | 95.51 | BRI1-associated receptor kinase |
|  |  |  | Glyma.05G119600 | 77.62 | BRI1-associated receptor kinase |
|  |  |  | Glyma.05G119700 | 62.44 | RNA-binding (RRM/RBD/RNP motifs) family protein with retrovirus zinc finger-like domain |
|  |  |  | Glyma.05G119800 | 55.19 | beta-D-xylosidase 4 |
|  |  |  | Glyma.05G119900 | 36.34 | Glycosyl hydrolase family protein |
|  |  |  | Glyma.05G120100 | 15.38 | loricrin-related |
|  |  |  | Glyma.05G120300 | 7.83 | FAD/NAD(P)-binding oxidoreductase family protein |
|  |  |  | Glyma.05G120400 | 5.70 | P-loop containing nucleoside triphosphate hydrolases superfamily protein |
|  |  |  | Glyma.05G120500 | 22.71 | xylem NAC domain 1 |
|  |  |  | Glyma.05G120600 | 29.63 | Mitochondrial substrate carrier family protein |
|  |  |  | Glyma.05G120700 | 37.74 | Zinc-binding ribosomal protein family protein |
|  |  |  | Glyma.05G120800 | 38.50 | ENTH/ANTH/VHS superfamily protein |
|  |  |  | Glyma.05G121000 | 51.50 | methyltransferases;nucleic acid binding |
|  |  |  | Glyma.05G121100 | 68.21 | RNA-binding (RRM/RBD/RNP motifs) family protein with retrovirus zinc finger-like domain |
|  |  |  | Glyma.05G121200 | 74.90 | Vacuolar iron transporter (VIT) family protein |
|  |  |  | Glyma.05G121300 | 81.49 | Vacuolar iron transporter (VIT) family protein |
|  |  |  | Glyma.05G121400 | 91.92 | Vacuolar iron transporter (VIT) family protein |
|  |  |  | Glyma.05G121500 | 96.56 | Vacuolar iron transporter (VIT) family protein |
| Gm05:31499885 | 5 | 31499885 | Glyma.05G035800 | 13.15 | RING/U-box superfamily protein |
| Gm06:42106014 | 6 | 42106014 | Glyma.06G248900 | 92.96 | NAC (No Apical Meristem) domain transcriptional regulator superfamily protein |
|  |  |  | Glyma.06G249100 | 26.44 | NAC domain containing protein 2 |
|  |  |  | Glyma.06G249300 | 9.73 | Cyclophilin-like peptidyl-prolyl cis-trans isomerase family protein |
|  |  |  | Glyma.06G249400 | 20.30 | S-adenosyl-L-methionine-dependent methyltransferases superfamily protein |
|  |  |  | Glyma.06G249500 | 31.38 | HAL2-like |
|  |  |  | Glyma.06G249700 | 93.79 | Late embryogenesis abundant (LEA) protein-related |
| Gm07:20934425 | 7 | 20934425 | Glyma.07G160800 | 46.62 | PIF1 helicase |
|  |  |  | Glyma.07G161000 | 17.01 | PPPDE putative thiol peptidase family protein |
|  |  |  | Glyma.07G161100 | 48.96 | WRKY family transcription factor family protein |
|  |  |  | Glyma.07G161200 | 62.28 | P-loop containing nucleoside triphosphate hydrolases superfamily protein |
|  |  |  | Glyma.07G161400 | 95.38 | NAP1-related protein 2 |
| Gm08:41958637 | 8 | 41958637 | Glyma.08G300500 | 93.15 | phytochrome kinase substrate 1 |
|  |  |  | Glyma.08G300700 | 81.50 | S-adenosyl-L-methionine-dependent methyltransferases superfamily protein |
|  |  |  | Glyma.08G300800 | 68.12 | CAAX amino terminal protease family protein |
|  |  |  | Glyma.08G300900 | 56.04 | open reading frame 204 |
|  |  |  | Glyma.08G301000 | 54.53 | NADH dehydrogenase 6 |
|  |  |  | Glyma.08G301100 | 45.48 | NAC (No Apical Meristem) domain transcriptional regulator superfamily protein |
|  |  |  | Glyma.08G301200 | 39.24 | Disease resistance protein (TIR-NBS-LRR class) family |
|  |  |  | Glyma.08G301300 | 26.75 | Ribosomal protein L34e superfamily protein |
|  |  |  | Glyma.08G301700 | 3.90 | Homeodomain-like superfamily protein |
|  |  |  | Glyma.08G301800 | 16.48 | NB-ARC domain-containing disease resistance protein |
|  |  |  | Glyma.08G301900 | 48.21 | Insulinase (Peptidase family M16) protein |
|  |  |  | Glyma.08G302000 | 58.45 | Regulator of chromosome condensation (RCC1) family with FYVE zinc finger domain |
|  |  |  | Glyma.08G302300 | 83.68 | Pentatricopeptide repeat (PPR) superfamily protein |
|  |  |  | Glyma.08G302400 | 87.75 | MIF4G domain-containing protein / MA3 domain-containing protein |
| Gm12:37109252 | 12 | 37109252 | Glyma.12G211600 | 72.41 | myb-like HTH transcriptional regulator family protein |
|  |  |  | Glyma.12G211800 | 33.08 | Protein kinase superfamily protein |
|  |  |  | Glyma.12G211900 | 24.68 | Peroxidase superfamily protein |
|  |  |  | Glyma.12G212000 | 12.23 | GSK3/SHAGGY-like protein kinase 1 |
|  |  |  | Glyma.12G212100 | 5.59 | DNAJ heat shock N-terminal domain-containing protein |
|  |  |  | Glyma.12G212300 | 21.90 | WRKY DNA-binding protein 69 |
|  |  |  | Glyma.12G212400 | 26.03 | Ras-related small GTP-binding family protein |
|  |  |  | Glyma.12G212500 | 41.43 | disease resistance protein (TIR-NBS-LRR class), putative |
|  |  |  | Glyma.12G212600 | 51.59 | UDP-Glycosyltransferase superfamily protein |
|  |  |  | Glyma.12G212700 | 58.65 | UDP-Glycosyltransferase superfamily protein |
|  |  |  | Glyma.12G212800 | 70.67 | Homeobox-leucine zipper family protein / lipid-binding START domain-containing protein |
| Gm13:9742355 | 13 | 9742355 | Glyma.13G030300 | 31.43 | lipoxygenase 2 |
| Gm17:38293751 | 17 | 38293751 | Glyma.17G227400 | 85.28 | IQ-domain 6 |
|  |  |  | Glyma.17G227500 | 83.03 | cytochrome P450, family 711, subfamily A, polypeptide 1 |
|  |  |  | Glyma.17G227600 | 55.82 | heat shock transcription factor A2 |
|  |  |  | Glyma.17G227700 | 34.73 | Thioredoxin superfamily protein |
|  |  |  | Glyma.17G227800 | 18.27 | beta-fructofuranosidase 5 |
|  |  |  | Glyma.17G227900 | 9.28 | 6-&1-fructan exohydrolase |
|  |  |  | Glyma.17G228000 | 3.62 | indeterminate(ID)-domain 2 |
|  |  |  | Glyma.17G228100 | 12.02 | FTSH protease 4 |
|  |  |  | Glyma.17G228300 | 22.60 | alpha/beta-Hydrolases superfamily protein |
|  |  |  | Glyma.17G228400 | 39.03 | Fatty acid/sphingolipid desaturase |
|  |  |  | Glyma.17G228500 | 49.81 | nucleotide binding protein 35 |
|  |  |  | Glyma.17G228600 | 55.94 | iron regulated transporter 3 |
|  |  |  | Glyma.17G228700 | 70.67 | cytokinin-responsive gata factor 1 |
|  |  |  | Glyma.17G228800 | 89.43 | glycine decarboxylase P-protein 2 |
| Gm18:14428467 | 18 | 14428467 | Glyma.18G116900 | 67.31 | alanine:glyoxylate aminotransferase |
|  |  |  | Glyma.18G117100 | 40.72 | Basic-leucine zipper (bZIP) transcription factor family protein |
|  |  |  | Glyma.18G117400 | 39.74 | MIF4G domain-containing protein / MA3 domain-containing protein |
|  |  |  | Glyma.18G117500 | 41.96 | MIF4G domain-containing protein / MA3 domain-containing protein |
|  |  |  | Glyma.18G117600 | 63.23 | Pentatricopeptide repeat (PPR) superfamily protein |
| Gm19:714816 | 19 | 714816 | Glyma.19G006500 | 96.91 | myb domain protein 21 |
|  |  |  | Glyma.19G006600 | 71.86 | Tudor/PWWP/MBT superfamily protein |
|  |  |  | Glyma.19G006700 | 57.24 | Adenine nucleotide alpha hydrolases-like superfamily protein |
|  |  |  | Glyma.19G006900 | 49.62 | NAD(P)-binding Rossmann-fold superfamily protein |
|  |  |  | Glyma.19G007200 | 28.26 | NAD(P)-binding Rossmann-fold superfamily protein |
|  |  |  | Glyma.19G007300 | 19.89 | Protein kinase superfamily protein |
|  |  |  | Glyma.19G007400 | 3.44 | ATPase E1-E2 type family protein / haloacid dehalogenase-like hydrolase family protein |
|  |  |  | Glyma.19G007600 | 24.95 | Protein of unknown function (DUF1645) |
|  |  |  | Glyma.19G007700 | 59.78 | carbonic anhydrase 1 |
|  |  |  | Glyma.19G007800 | 69.34 | DegP protease 1 |
|  |  |  | Glyma.19G008100 | 89.56 | GDSL-like Lipase/Acylhydrolase superfamily protein |
|  |  |  | Glyma.19G008200 | 95.88 | Tetratricopeptide repeat (TPR)-like superfamily protein |
| Gm19:839834 | 19 | 839834 | Glyma.19G007700 | 65.24 | carbonic anhydrase 1 |
|  |  |  | Glyma.19G007800 | 55.68 | DegP protease 1 |
|  |  |  | Glyma.19G008100 | 35.46 | GDSL-like Lipase/Acylhydrolase superfamily protein |
|  |  |  | Glyma.19G008200 | 29.14 | Tetratricopeptide repeat (TPR)-like superfamily protein |
|  |  |  | Glyma.19G008300 | 19.40 | Oxidoreductase, zinc-binding dehydrogenase family protein |
|  |  |  | Glyma.19G008400 | 7.09 | Oxidoreductase, zinc-binding dehydrogenase family protein |
|  |  |  | Glyma.19G008500 | 2.19 | Oxidoreductase, zinc-binding dehydrogenase family protein |
|  |  |  | Glyma.19G008600 | 2.12 | Oxidoreductase, zinc-binding dehydrogenase family protein |
|  |  |  | Glyma.19G008700 | 8.08 | Oxidoreductase, zinc-binding dehydrogenase family protein |
|  |  |  | Glyma.19G008800 | 13.16 | Uncharacterised protein family (UPF0497) |
|  |  |  | Glyma.19G008900 | 17.83 | Prenyltransferase family protein |
|  |  |  | Glyma.19G009000 | 21.14 | formate dehydrogenase |
|  |  |  | Glyma.19G009100 | 26.75 | formate dehydrogenase |
|  |  |  | Glyma.19G009300 | 41.47 | Pre-rRNA-processing protein TSR2, conserved region |
|  |  |  | Glyma.19G009400 | 45.21 | HD domain-containing metal-dependent phosphohydrolase family protein |
|  |  |  | Glyma.19G009500 | 57.91 | Protein kinase superfamily protein |
|  |  |  | Glyma.19G009700 | 67.80 | cyclophilin 38 |
|  |  |  | Glyma.19G009800 | 77.93 | Nodulin MtN3 family protein |
|  |  |  | Glyma.19G009900 | 83.22 | Nodulin MtN3 family protein |

**Table S4.** Cis-acting elements prediction for 2.0 kb flanking region of candidate genes.

| Gene ID | Site Name | Position(bp) | Strand | Sequence | Function |
| --- | --- | --- | --- | --- | --- |
| Glyma.05G121100 | ABRE | 1971 | + | AACCCGG | cis-acting element involved in the abscisic acid responsiveness |
|  | TCA-element | 1706 | + | CCATCTTTTT | cis-acting element involved in salicylic acid responsiveness |
| Glyma.08G301200 | O2-site | 342 | - | GATGA(C/T)(A/G)TG(A/G) | cis-acting regulatory element involved in zein metabolism regulation |
|  | WUN-motif | 37 | - | AAATTTCCT | wound-responsive element |
| Glyma.17G228300 | TC-rich repeats | 1940 | + | ATTCTCTAAC | cis-acting element involved in defense and stress responsiveness |
| Glyma.19G006900 | TCA-element | 1841 | - | CCATCTTTTT | cis-acting element involved in salicylic acid responsiveness |
|  | TGACG-motif | 1934 | + | TGACG | cis-acting regulatory element involved in the MeJA-responsiveness |
| Glyma.19G008700 | TCA-element | 1481 | - | TCAGAAGAGG | cis-acting element involved in salicylic acid responsiveness |
|  | TCA-element | 1976 | + | CCATCTTTTT | cis-acting element involved in salicylic acid responsiveness |
| Glyma.19G008900 | TC-rich repeats | 199 | - | ATTCTCTAAC | cis-acting element involved in defense and stress responsiveness |
|  | TCA-element | 1075 | + | CCATCTTTTT | cis-acting element involved in salicylic acid responsiveness |
